# L-Tryptophan Differentially Regulated Glucose and Amino Acid Transporters in the Small Intestine of Rat Challenged with Lipopolysaccharide

**DOI:** 10.3390/ani12213045

**Published:** 2022-11-05

**Authors:** Bin Wang, Lili Jiang, Zhenlong Wu, Zhaolai Dai

**Affiliations:** State Key Laboratory of Animal Nutrition, College of Animal Science and Technology, China Agricultural University, Beijing 100193, China

**Keywords:** L-tryptophan, amino acid transporter, lipopolysaccharide, Ussing chamber, rat

## Abstract

**Simple Summary:**

This study aimed to test the hypothesis that L-tryptophan (Trp) modulates lipopolysaccharide (LPS)-induced changes in glucose and amino acid (AA) transport in the rat jejunum in a transporter-dependent manner. Rats were fed Trp in drinking water for 7 days and were intraperitoneally injected with LPS once. Jejunum samples isolated from rats were used for the determination of glucose and AA transport using an Ussing chamber and the expression of related transporters. We found that LPS-induced gut inflammation reduced glucose and AA transport in the jejunum of the rats. Tryptophan supplementation alleviated the LPS-induced downregulation of the expression of the glucose transporter *SGLT1*, the AA transporters solute carrier family 38 member 2 (*SNAT2*) and solute carrier family 7 member 8 (*LAT2*), as well as ATPase Na^+^/K^+^ transporting subunit alpha 2 (*ATP1A2*); however, it increased the LPS-induced upregulation of acidic AA transporter solute carrier family 1 member 1 (*EAAT3*) expression.

**Abstract:**

Tryptophan (Trp) has been shown to improve the growth and gut function of weaned piglets. Whether the growth-promoting effect of Trp is due to the improvement in nutrient transport and absorption during weaning or under conditions of inflammation has not been fully characterized. The objective of this study was to determine the effects of Trp on lipopolysaccharide (LPS)-induced changes in glucose and amino acid (AA) transport in the rat jejunum. Twenty-four 7-week-old Sprague Dawley rats were randomly divided into one of three groups: control, LPS, and Trp + LPS. Rats were supplemented with 0 or 0.1 mg Trp per gram body weight/d in drinking water for 7 days and were intraperitoneally injected with LPS (5 mg/kg BW) on day 8. After 24 h, rats were sacrificed, and jejunum samples were isolated for the analysis of glucose and AA transport using an Ussing chamber and the expression of glucose and AA transporters. The results showed that Trp alleviated the LPS-induced increase in jejunal permeability (*p* < 0.05) and decrease in changes in the short-circuit current of glucose, arginine, glutamine, glutamate, glycine, histidine, leucine, lysine, taurine, threonine, and Trp (*p* < 0.05). Trp reversed (*p* < 0.05) the LPS-induced downregulation of expression of the glucose transporter *SGLT1* and AA transporters solute carrier family 38 member 2 (*SNAT2*) and solute carrier family 7 member 8 (*LAT2*), as well as ATPase Na^+^/K^+^ transporting subunit alpha 2 (*ATP1A2*). However, Trp increased (*p* < 0.01) the LPS-induced upregulation of acidic AA transporter solute carrier family 1 member 1 (*EAAT3*) expression. The above findings may help to develop nutritional interventions for the differential targeting of gut nutrient transporters, aiming to improve gut function and health in the presence of inflammation in both humans and animals.

## 1. Introduction

Nutrient transport is important for the digestion and absorption of dietary components in the intestine and plays a crucial role in the growth and maturation of gut epithelial cells [1,2]. The transport of glucose and amino acid (AA) in the small intestine is carried out by a variety of transporters [3,4]. Factors such as dietary composition, gut development, and gut inflammation have been shown to regulate the activity of these transporters and to affect nutrient absorption, gut function, and growth [5].

Studies on the developmental changes and dietary regulation of the intestinal AA transporters in animals showed that the expression of the sodium-dependent neutral amino acid transporter 2 (solute carrier family 38, *SNAT2*) in the basolateral membrane of the jejunal mucosa decreased one day after weaning [6]. Dietary supplementation with an AA blend containing glutamine, glutamate, glycine, arginine, and *N*-acetylcysteine increased the expression of solute carrier family 6 member 19 (*b*^0^^,*+*^*AT*) and solute carrier family 7 member 7 (*y^+^LAT1*) in the jejuna of weaning piglets [7]. An increased concentration of glutamate in the culture medium upregulated the expression of solute carrier family 1 member 1 (*EAAT3*) in piglet enterocytes [8]. It was found that, compared to a normal-protein diet, a high-protein diet or AA diet upregulated the expression of solute carrier family 6 member 19 (*B*^0^*AT1*) in the proximal jejuna rather than in the distal jejuna of rats [9]. When exposed to heat stress, pigs fed a low-protein diet supplemented with free AAs expressed a lower level of *y^+^LAT1* in the jejunum compared with the pigs fed a high-protein diet [10].

Inflammation has been shown to regulate glucose and AA transporters in the intestine. Microarray-based analysis showed that piglets infected with porcine epidemic diarrhea virus (PEDV) showed downregulated expression of AA transporters such as *y^+^LAT1*, *EAAT3*, *b*^0^^,*+*^*AT*, solute carrier family 15 member 1 (*PepT1*), solute carrier family 1 member 4 (*ASCT1*), and solute carrier family 3 member 1 (*rBAT*); however, they showed upregulated expression of *B*^0^*AT1* in the jejunum [11]. Additionally, PEDV infection downregulated glucose transporter-2 (*GLUT2*) expression and upregulated sodium/glucose co-transporter-1 (*SGLT1*) expression [11]. A study using rabbits showed that lipopolysaccharide (LPS) challenge decreased the mucosal to serosal transepithelial flux and the transport of L-leucine across the brush border membrane of the jejunum [12]. Recent studies have suggested that angiotensin-converting enzyme 2 (ACE2) is the receptor of the coronavirus SARS-CoV-2 and is related to the expression of AA transporters on the surface of small intestinal mucosal cells that are important in the regulation of transport of AAs into the cytosol of enterocytes, and therefore in the regulation of gut immune function [13]. Recent studies using the porcine intestinal cell line IPEC-J2 have shown that the epidermal growth factor promotes AA and glucose absorption via the upregulation of *SGLT1* and *GLUT2* expression [14,15].

Tryptophan (Trp) is an essential AA in mammals and has been shown to regulate body immunity through the production of versatile metabolites that participate in the regulation of immune-related signaling pathways [5,16]. Our previous study showed that dietary supplementation with Trp improved the growth and alleviated the gut immune response of piglets 30 days after weaning [17]. Additionally, increased levels of Trp in the diet altered the concentrations of free AAs such as glutamate, taurine, and Trp in the blood of piglets [18]. A study using the intestinal porcine epithelial cell line IPEC-1 showed that Trp regulated the expression of the AA transporters *B*^0^*AT1*, solute carrier family 6 (neurotransmitter transporter), member 14 (*ATB*^0^^,*+*^), and solute carrier family 3 member 1 (*rBAT*), as well as ATPase Na^+^/K^+^ transporting subunit alpha 1 (*ATP1A1*) [19]. The above findings suggest that dietary Trp supplementation partially alleviated weaning stress and gut immune response and improved animal growth by modulating gut nutrient transport for whole-body use. However, the characteristics and mechanism of dietary Trp in the regulation of nutrient transport in the small intestine in the context of inflammation is not yet clear.

This study aimed to test the hypothesis that dietary tryptophan supplementation before the onset of the gut immune response induced by LPS differentially regulates the expression of glucose and AA transporters and improves nutrient transport in the jejuna of rats. The findings of this study will help to explain the underlying mechanism of the improvement in gut function and animal growth following tryptophan supplementation and provide a basis for the development of nutritional interventions for improving the enteral nutrition of the inflamed gut.

## 2. Materials and Methods

### 2.1. Animals and Diets

Twenty-four 7-week-old Sprague Dawley rats (purchased from Beijing Vital River Laboratory Animal Technology, Beijing, China) with an average body weight (BW) of 191 g were maintained in standard rat cages under specific pathogen-free conditions in an animal facility at China Agricultural University. Rats were housed in an environment that was maintained at 22–25 °C and that had 45–55% relative humidity and a 12 h light/dark cycle, and had free access to feed and water (drinking water or Trp solution, depending on treatment). The standard rodent diet used in the experiment provided the following nutrients: 20% crude protein, 4% crude fat, 5% crude fiber, 1.4% calcium, and 0.9% total phosphorus (catalogue number 1032, Beijing HFK Bioscience Co., Beijing, China). The Trp content in the diet was 0.19%. All animal experiments were approved by the Institutional Animal Care and Use Committee of China Agricultural University (AW52101202-2-4).

### 2.2. Experimental Design and Sample Collection

Rats were randomly divided into three groups: control, LPS (L2637, Merck, Shanghai, China), and Trp + LPS. All rats were weighed on day 0 and day 7 of the experiment, and body weight (BW), feed intake, and water consumption were recorded. Rats had free access to drinking water or Trp solution (0.6 g Trp/L drinking water, providing 0.1 mg/(g BW·d)) throughout the entire experimental period. Drinking water was changed daily. The total daily Trp intake was 62% greater in the Trp group than in the control group (Table 1). The dose of Trp we used in the current study was based on our previous study with mice, which showed that a dietary Trp level set to 150% compared to the Trp level of the control diet alleviated dextran sodium sulfate-induced gut inflammation and weight loss in mice [20]. On day 8, rats in the LPS and Trp + LPS groups were intraperitoneally injected with 5 mg/kg BW of LPS. Rats in the control group were intraperitoneally injected with an equal volume of sterile 0.9% saline.

On day 9 of the experiment, all of the rats had fasted for 12 h and were sacrificed by cervical dislocation. The jejunum was isolated, and a portion of the jejunum that was 10 cm in length was taken 5 cm from the end of the duodenum. After sampling, the gut segment was placed in Krebs–Henseleit bicarbonate buffer (KHB buffer, pH = 7.4) and flushed with 95% O_2_/5% CO_2_ gas in a 37 °C water bath for Ussing chamber analysis. Another 5 cm portion of jejunum was snap-frozen in liquid nitrogen and stored at −80 °C for further analysis.

### 2.3. Ussing Chamber Analysis

The transepithelial resistance (R_t_), conductance (G_t_), and short-circuit current (I_sc_) of the jejunum was measured using the 6-channel Ussing chamber system (MC6-6, Physiologic Instrument, Reno, NV, USA). A mid-portion of the jejunum from each rat that was 10 cm in length was opened and flushed with prechilled KHB buffer to remove any remaining luminal content. The jejunum was then quickly divided into segments that were 1 cm in length, which were then pinned onto 0.5 cm^2^ slides and then mounted into the Ussing chamber within 30 min after sampling. The mucosal side and serosal side of the jejunum were filled with 5 mL of KHB buffer. The system was maintained at 37 °C via constant water bath circulation and oxygenated with a 95% O_2_/5% CO_2_ gas. After the I_sc_ of the jejunum was stabilized, 0.5 mL of 50 mM AA solution (final concentration 5 mM) was added to the chambers on the mucosal side, and 0.5 mL of 50 mM mannitol solution (final concentration 5 mM) was added to the chambers on the serosal side. The R_t_, G_t_ and I_sc_ were continuously recorded using Acquire & Analyze software (Physiologic Instruments, Reno, NV, USA).

### 2.4. Quantitative RT-PCR

Total RNA was isolated from the jejunum samples using TRIzon reagent. Reverse transcription was performed using the PrimerScript RT Reagent Kit (TaKaRa, Beijing, China). Real-time PCR was performed using the SYBR Premix Ex Taq II (TaKaRa) on the Applied Biosystems 7500 Sequence Detection System (Applied Biosystems, Waltham, MA, USA). The primer sequences used for the quantification of inflammatory cytokines, glucose and amino acid transporters, and Na^+^/K^+^-ATPase are listed in Table 2. Glyceraldehyde-3-phosphate dehydrogenase (*GAPDH*) was used as the internal reference. Results were calculated using the 2^−ΔΔCt^ method [20].

### 2.5. Statistical Analysis

Values are presented as means ± SEM. Data were analyzed by one-way ANOVA and the Tukey multiple-comparison procedure in SAS (version 9.0, SAS Institute Inc., Cary, CA, USA). *p*-Values < 0.05 were considered statistically significant.

## 3. Results

### 3.1. Animal Growth and Expression of Inflammatory Cytokines in the Jejunum

**Figure 1:**
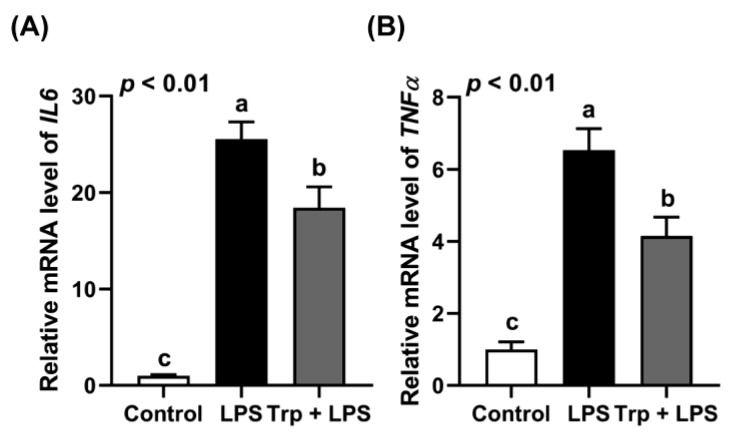
Effect of tryptophan supplementation on the expression of *IL6* (**A**) and *TNFα* (**B**) in the jejuna of LPS-challenged rats. Values are means ± SEM, *n* = 6–8; a–c: values without a common letter differ (*p* < 0.01).

Before the LPS challenge, Trp supplementation in drinking water for 7 days did not alter the growth and feed intake of the rats (*p* > 0.05) (Table 3). Trp supplementation alleviated the LPS-induced upregulation of *IL6* and *TNFα* expression in the jejuna of the rats (Figure 1).

### 3.2. Effects of Trp and LPS on Transepithelial Resistance and Conductance of Rat Jejunum

**Figure 2:**
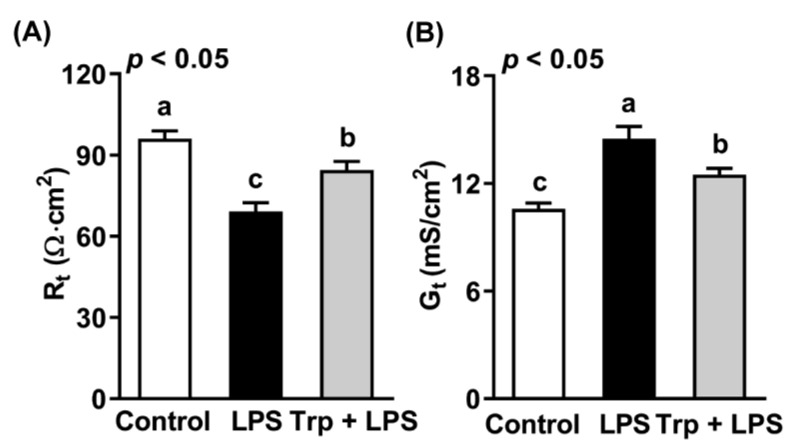
Effect of tryptophan supplementation on transepithelial resistance (**A**) and conductance (**B**) of the jejuna of LPS-treated rats. Values are means ± SEM, *n* = 8; a–c: values without a common letter differ (*p* < 0.05). R_t_, transepithelial resistance; G_t_, transepithelial conductance.

The jejuna of rats from different treatment groups were subjected to measurement of transepithelial resistance (R_t_) and conductance (G_t_) using an Ussing chamber (Figure 2). Compared with the control group, the intraperitoneal injection of LPS reduced R_t_ by 28% (Figure 2A) and increased G_t_ by 37% (Figure 2B) (*p* < 0.05). Trp supplementation before LPS treatment alleviated LPS-induced changes in R_t_ and G_t_ in the jejuna of rats (*p* < 0.05). The above data indicate that Trp alleviated the LPS-induced increase in permeability and maintained the gut barrier of the rat jejunum.

### 3.3. Effects of Trp on LPS-Induced Changes in Short-Circuit Current of the Rat Jejunum in Response to Glucose and AAs

As revealed by changes in the short-circuit current (ΔI_sc_), the LPS challenge reduced (*p* < 0.05) the sodium-ion-dependent transport of glucose and AAs tested by the rat jejunum compared to the control (Table 4). Trp supplementation before LPS treatment alleviated the LPS-induced decrease in ΔI_sc_ of glucose, Arg, Gln, Glu, Leu, Thr and Trp (*p* < 0.05).

### 3.4. Effects of Trp and LPS on Gene Expression of Glucose and Amino Acid Transporters in Rat Jejunum

Compared to the control, LPS treatment reduced (*p* < 0.05) the expression of sodium-ion-dependent glucose (*SGLT1*) and neutral amino acid transporters (*SNAT2* and *LAT2*), as well as that of ATPase Na^+^/K^+^ transporting subunit alpha 2 (*ATP1A2*) (Table 5). However, the expression of acidic and basic amino acid transporters (*EAAT3*, *ATB*^0^^,*+*^, and *y^+^LAT2*) was upregulated (*p* < 0.05) in the jejuna of rats challenged with LPS (Table 5). Trp supplementation before LPS treatment attenuated (*p* < 0.05) the downregulation of *SGLT1*, *SNAT2*, *LAT2*, and *ATP1A2* expression (Table 5). Moreover, Trp treatment promoted the LPS-induced upregulation of *EAAT3* expression (*p* < 0.01) (Table 5). However, Trp supplementation did not alter the LPS-induced upregulation of *ATB*^0^^,*+*^ or *y^+^LAT2* expression (Table 5).

## 4. Discussion

Inflammation of the intestine induced by infection and weaning stress not only leads to immune response but also affects the gut barrier and nutrient transport and absorption [21]. This, in turn, affects the outcomes of immunity-related gut function in the presence of inflammation [22]. Levels of endotoxins such as LPS from the cell walls of Gram-negative bacteria are relatively low in the blood of healthy humans and animals; however, increasing numbers of these bacteria in the blood during states of gut inflammation can cause tissue damage and nutrient malabsorption in the intestine [23]. Dietary factors such as functional AAs have been proven to improve gut barrier function and nutrient absorption, and therefore may regulate AA transport in the intestine during inflammation [5]. Additionally, our study on piglets showed that dietary Trp improved gut tight-junction function, feed utilization, and animal growth after weaning through the regulation of nutrient absorption [17,18]. Although an attempt was previously made to test the effects of mixtures of aromatic amino acids on the expression of AA transporters in the small intestine as well as on concentrations of free AAs in the plasma of piglets challenged with LPS, their effects on the changes in plasma free AA concentrations were complex and require further validation [24]. Additionally, the effect of dietary supplementation with Trp on the transport of specific AAs in the small intestine under conditions of inflammation requires further investigation. Findings in this area will help us to better understand the mechanism of functional AAs on the regulation of nutrient transport and absorption under conditions of gut inflammation and to develop new dietary strategies for improving nutrient absorption and gut function during the onset of gut inflammation.

This study showed that LPS challenge increased permeability and reduced the transport of some essential and functional AAs in the jejuna of rats. A similar observation was found in a study using rabbits, which showed that the effect of LPS was due to a reduction in the apparent transport and activity capacity of Na^+^/K^+^ ATPase [12]. Additionally, LPS challenge reduced the transepithelial electrical resistance of the jejunum in weaned piglets [25]. Therefore, the downregulation of *ATP1A2* expression by LPS in our current study may have reduced sodium transport and affected the activity of sodium-dependent AA transport. Moreover, one should bear in mind that the absorption of dietary glucose and AAs into circulation occurs via the combination of transcellular active transport and paracellular transport [26]. LPS-induced defects in the glucose and AA transporters of the enterocytes will hinder recovery from inflammation-induced damage; however, increased gut permeability may increase the paracellular transport of AAs and increase peripheral levels of AA during gut inflammation as result [26].

Another important finding of this study is that the LPS-induced defects in glucose transport may be due mainly to the downregulation of the glucose transporter *SGLT1* at the bush border rather than at the basolateral border (*GLUT2*) of the small intestine. Due to the affinity of *SGLT1* to glucose being high [3], the alleviation of LPS-induced defects in glucose transport by Trp may have important implications for improving glucose absorption during weaning stress and gut inflammation. It has been found that the immune response is energetically costly and that the function of gut immune cells is influenced by many dietary nutrients [5,22,27]. Moreover, a recent study using weaning piglets suggested that glutamine, glutamate, and aspartate differentially regulated the energy metabolism and homeostasis of the small intestine through the replenishment of the Krebs’ cycle and the modulation of adenosine monophosphate-activated protein kinase (AMPK) signaling [28]. However, it is not clear whether the energy deficiency modulated AA transport or vice versa. Further studies are warranted to uncover the interactions of sugars and AAs as well as of glucose and AA transporters in the transport and absorption of nutrients in gut health and disease [29].

Our current study also suggested that dietary supplementation of Trp before the onset of the immune response of the small intestine to LPS alleviated AA transport by differentially regulating the expression of related transporters in the rat jejunum. For the transport of neutral AAs such as glutamine, glycine, leucine, threonine, and Trp, LPS upregulated the expression of the apical neutral AA transporter *ATB*^0^^,*+*^; however, it reduced the expression of the basolateral neutral AA transporter *LAT2* in the jejunum. This may have led to the accumulation of these AAs in the intestine for local use in the immune response rather than to them being transported into the bloodstream. Trp supplementation before LPS challenge alleviated this effect. A study considering the difference in the expression and protein abundance of the AA transporters *LAT1* and *LAT2* in the intestine showed that the expression of *LAT2* is higher than that of *LAT1* in the jejunal mucosa of rats, while the protein abundance of LAT1 is higher in the rat colon [30]. The above observations suggest that the response of AA transporters to intestinal inflammation is gut-segment-specific. Additionally, for the transport of alanine, glutamine, histidine, and methionine, LPS challenge downregulated *SNAT2* and upregulated basolateral *y^+^LAT2* expression. It was found that the expression of *SNAT2* in the jejunum and ileum was decreased one day after weaning in piglets and under AA starvation in enterocytes [6]. Therefore, reducing the activity of SNAT2 and increasing the activity of y^+^LAT2 in the enterocytes of the jejunum may cause starvation of the above-mentioned AAs in cells. Furthermore, the expression of the aspartate and glutamate transporter *EAAT3* was upregulated by LPS challenge and Trp supplementation before the onset of gut inflammation dramatically increased *EAAT3* expression. A study regarding the expression of *EAAT3* in the intestines of turkey and mice showed that the expression of *EAAT3* was higher than that of other AA transporters and that the expression was gut-segment-specific in the small intestine [31,32]. The above findings suggest that the response of *EAAT3* to luminal stimuli such as AAs in the small intestine may be sensitive to other AA transporters. Moreover, although the expression of the glutamate transporter *EAAT3* was dramatically upregulated, the short-circuit current of the jejunum did not change much in response to the addition of glutamate to the mucosal side. A possible explanation is that the protein abundance and functionality of EAAT3 in response to LPS treatment and Trp supplementation was delayed compared to its gene expression, and further studies to quantify the protein abundance of the AA transporters in the small intestine are warranted.

Additionally, it was found that glutamine, glutamate, and aspartate are the major energy sources in the enterocytes of piglets [33]. Due to a defect in glucose transport at the apical membrane, increasing the levels of glutamate and aspartate transport into the enterocytes will alleviate increased energy expenditure. The upregulation of glutamate and aspartate plus glucose transport by Trp before gut inflammation can be regarded as one of the regulatory roles and complementary strategies of the intestine in combating inflammation and in facilitating gut repair. However, further studies are warranted to confirm these hypotheses as well as to uncover the underlying mechanisms. Factors such as type of infection (e.g., bacterial or viral), site of infection in the gastrointestinal tract, cell-type-specific AA transport and metabolism of immune cells, and nutritional background should not be neglected [22,34,35].

## 5. Conclusions

In conclusion, LPS-induced gut inflammation regulated glucose and AA transport in the jejuna of rats. Tryptophan supplementation before the onset of the gut immune response differentially altered the expression of glucose and AA transporter genes and alleviated the changes in glucose and AA transport as a result. Considering the multifunctionality and compartmentation of both the AA transporters and their regulators/stabilizers (e.g., ACE2) in the transport and absorption of AAs and other compounds in the small intestine, it is important to uncover the characteristics and mechanisms of the regulatory role of tryptophan in AA transport along the gastrointestinal tract in terms of the energy and nitrogen homeostasis of the body. The interactions among nutrients, gut microbiota, and host should be taken into consideration for the development of personalized nutritional interventions for the differential regulation of nutrient absorption and gut health in both humans and animals.

## Figures and Tables

**Table 1:** Daily intake of tryptophan by rats in control and Trp groups before LPS treatment ^1^.

Treatments	Trp Intake from Drinking Water (mg/d)	Trp Intake from Feed (mg/d)	Total Daily Trp Intake (mg/d)	Trp Intake, % of Control	
				Feed	Total
Control	0.00 ± 0.00	45.2 ± 3.00	45.2 ± 3.00	100	100
Trp	25.06 ± 1.98	47.9 ± 2.23	73.0 ± 2.79	106	162

^1^ Values are means ± SEM, *n* = 8.

**Table 2:** Primers used in this study.

Genes		Primer Sequence (5′ → 3′)	Product Size, bp
*IL6*	Forward	CAAGAGACTTCCAGCCAGTTG	106
	Reverse	TGGGTGGTATCCTCTGTGAAG	
*TNFα*	Forward	GACTCTGACCCCCATTACTCTG	151
	Reverse	GTCTCGTGTGTTTCTGAGCATC	
*ATB*^0^^,*+*^ (*Slc6a14*)	Forward	TATCCGGAAGCACTAGCTCAA	82
	Reverse	CCAGACCCAATGTTAAAAGCA	
*ATP1A1*	Forward	TAAAGTGCATCGAGGTCTGCT	121
	Reverse	TTTGGGTTCTTGTGAATGGAG	
*ATP1A2*	Forward	GGGAACATGCAGAATCAGTGT	112
	Reverse	CCAGCTTCCTGTCTCAATCTG	
*B*^0^*AT1* (*Slc6a19*)	Forward	GGACATATCTCATCGCCTTCA	162
	Reverse	CGATGTCCTTGTTGAACCTGT	
*EAAT3* (*Slc1a1*)	Forward	GGGCTTCTCGGTAGACAAATC	151
	Reverse	TTGTGTCCTTCTCGCTCCTTA	
*GLUT2* (*Slc2a2*)	Forward	CTCATCATTGCTGGAAGAAGC	133
	Reverse	CAAGAGCCAGTTGGTGAAGAG	
*LAT2* (*Slc7a8*)	Forward	CACATTTGGTGGAGTCAACG	105
	Reverse	TTCACGTGGATCATGGCTAA	
*SGLT1* (*Slc5a1*)	Forward	CCAACTTTGGTTTCTGATGGA	84
	Reverse	GTCTCGGAATATGTGGAACGA	
*SNAT2* (*Slc38a2*)	Forward	TCCTTCCGTCTGCTTTCTACA	78
	Reverse	AACACAGAGCCCCAATCTTTT	
*y^+^LAT1* (*Slc7a7*)	Forward	TCAGCTTTACCTACGCTGGAA	101
	Reverse	CCACCAGGAAGATGGTACAGA	
*GAPDH*	Forward	CTGTGACTTCAACAGCAACTCC	123
	Reverse	ACCCTGTTGCTGTAGCCATATT	

*ATB*^0,+^, solute carrier family 6 member 14; *ATP1A1*, ATPase Na^+^/K^+^ transporting subunit alpha 1; *ATP1A2*, ATPase Na^+^/K^+^ transporting subunit alpha 2; *B*^0^*AT1*, solute carrier family 6 member 19; *EAAT3*, solute carrier family 1 member 1; *GAPDH*, glyceraldehyde-3-phosphate dehydrogenase; *GLUT2*, solute carrier family 2 member 2; *IL6*, interleukin 6; *LAT2*, solute carrier family 7 member 8; *SGLT1*, solute carrier family 5 member 1; *SNAT2*, solute carrier family 38 member 2; *TNFα*, tumor necrosis factor α; *y^+^LAT1*, solute carrier family 7 member 7.

**Table 3:** Effect of tryptophan supplementation on the growth of rats before LPS challenge ^1^.

Items	Treatment		*p*-Value
	Control	Trp	
Body weight (g)			
d 0	218 ± 7.7	222 ± 6.8	0.70
d 7	248 ± 8.1	255 ± 6.7	0.51
Average daily weight gain (g/day)			
d 0–7	19.1 ± 1.3	21.6 ± 1.6	0.24
Average daily feed intake (g/day)			
	23.8 ± 2.18	25.2 ± 1.61	0.85

^1^ Values are means ± SEM, *n* = 8.

**Table 4:** Effects of tryptophan supplementation and LPS on changes of short-circuit current (ΔI_sc_) across the jejuna of rats ^1^.

Nutrient Added	Control	LPS	Trp + LPS	*p*-Value
Glucose	0.97 ± 0.10 ^a^	0.35 ± 0.04 ^b^	0.84 ± 0.10 ^a^	*p* < 0.05
Arg	1.29 ± 0.15 ^a^	0.36 ± 0.06 ^b^	1.06 ± 0.13 ^a^	*p* < 0.05
Gln	0.91 ± 0.14 ^a^	0.35 ± 0.08 ^b^	0.90 ± 0.17 ^a^	*p* < 0.05
Glu	0.57 ± 0.09 ^a^	0.14 ± 0.06 ^b^	0.40 ± 0.04 ^a^	*p* < 0.05
Gly	0.31 ± 0.04 ^a^	0.10 ± 0.03 ^b^	0.20 ± 0.06 ^ab^	*p* < 0.05
His	0.58 ± 0.04 ^a^	0.23 ± 0.13 ^b^	0.47 ± 0.07 ^ab^	*p* < 0.05
Leu	0.44 ± 0.02 ^a^	0.14 ± 0.02 ^b^	0.39 ± 0.06 ^a^	*p* < 0.05
Lys	0.55 ± 0.16 ^a^	0.12 ± 0.07 ^b^	0.44 ± 0.06 ^ab^	*p* < 0.05
Tau	0.44 ± 0.12 ^a^	0.11 ± 0.04 ^b^	0.34 ± 0.05 ^ab^	*p* < 0.05
Thr	0.88 ± 0.10 ^a^	0.10 ± 0.03 ^c^	0.55 ± 0.09 ^b^	*p* < 0.05
Trp	0.84 ± 0.14 ^a^	0.31 ± 0.08 ^b^	0.73 ± 0.12 ^a^	*p* < 0.05

^1^ ΔI_sc_ (μA/cm) = I_sc_ induced by glucose or amino acids -baseline I_sc_. Values are means ± SEM, *n* = 8; a–c: values in a row without a common letter differ (*p* < 0.05).

**Table 5:** Effect of tryptophan supplementation on LPS-induced changes in expression of nutrient transporters ^1^.

Genes (Fold of Control)	Control	LPS	Trp + LPS	*p*-Value
*GLUT2*	1.00 ± 0.13	1.49 ± 0.22	0.92 ± 0.19	0.16
*SGLT1*	1.00 ± 0.07 ^a^	0.38 ± 0.10 ^b^	0.94 ± 0.14 ^a^	*p* < 0.05
*SNAT2*	1.00 ± 0.11 ^ab^	0.56 ± 0.09 ^b^	1.39 ± 0.17 ^a^	*p* < 0.05
*LAT2*	1.00 ± 0.10 ^ab^	0.51 ± 0.06 ^b^	1.22 ± 0.17 ^a^	*p* < 0.05
*B* ^0^ *AT1*	1.00 ± 0.20	0.90 ± 0.19	1.20 ± 0.24	0.63
*EAAT3*	1.00 ± 0.27 ^c^	13.3 ± 1.8 ^b^	347 ± 7.2 ^a^	*p* < 0.01
*y^+^LAT2*	1.00 ± 0.18 ^b^	3.34 ± 0.26 ^a^	2.63 ± 0.25 ^a^	*p* < 0.01
*ATB* ^0^^,*+*^	1.00 ± 0.13 ^b^	23.9 ± 3.0 ^a^	17.4 ± 1.9 ^a^	*p* < 0.05
*ATP1A1*	1.00 ± 0.07	1.28 ± 0.10	1.18 ± 0.16	0.48
*ATP1A2*	1.00 ± 0.16 ^a^	0.07 ± 0.05 ^c^	0.40 ± 0.05 ^b^	*p* < 0.01

^1^ Values are means ± SEM, *n* = 6–8; a–c: values in a row without a common letter differ (*p* < 0.05).

## Data Availability

All data presented in the study were included in the manuscript.
